# Berubicin Substituted for Cytarabine in a MATRix-Based Regimen for Isolated Central Nervous System Lymphoma: A Five-Patient Case Series from the BER-PUM Study

**DOI:** 10.3390/curroncol33090557

**Published:** 2026-09-15

**Authors:** Sławomir Milczarek, Oliwia Piotrowska, Bartłomiej Baumert, Ewa Borowiecka, Krzysztof Sommerfeld, Alina Hnatyszyn, Bogusław Machaliński

**Affiliations:** Department of Hematology and Transplantology, Pomeranian Medical University, 71-252 Szczecin, Poland

**Keywords:** berubicin, primary central nervous system lymphoma, secondary central nervous system lymphoma, high-dose methotrexate, thiotepa, autologous stem-cell transplantation, dose escalation

## Abstract

Primary and isolated secondary central nervous system lymphomas are aggressive malignancies requiring effective central nervous system–directed therapy. Intensive high-dose methotrexate–based regimens may be difficult to deliver, particularly in older or functionally impaired patients. Berubicin is a doxorubicin analog developed to cross the blood–brain barrier. We report a prospective case series of five adults with primary or isolated secondary CNS lymphoma treated within the BER-PUM phase Ib/II study (EudraCT 2021-006028-41), evaluating berubicin as part of the investigational MBTRIX regimen. Patients received up to four 21-day cycles of rituximab, high-dose methotrexate, berubicin, and thiotepa, with between-patient and intrapatient berubicin dose escalation. Four out of five patients completed all planned cycles, even though most of the patients had an ECOG score of 2. After two cycles, four achieved partial response, and one had progressive disease. Two patients underwent autologous stem-cell transplantation; one achieved complete response maintained at 12 months, whereas the other died from severe infectious and multiorgan complications. All patients experienced at least one grade ≥ 3 adverse event, predominantly hematologic. No protocol-defined dose-limiting toxicity or berubicin dose reduction occurred. Recruitment ended prematurely because berubicin became unavailable. This case series demonstrates the feasibility of MBTRIX, although the small sample and incomplete dose escalation preclude definitive conclusions regarding safety and efficacy.

## Simple Summary

Lymphomas involving the central nervous system remain therapeutically challenging because many anticancer agents achieve inadequate concentrations within the brain. High-dose methotrexate-based regimens may provide clinical benefit but are associated with substantial toxicity, particularly in older or functionally impaired patients. We report a prospective case series of five patients treated with MBTRIX, an investigational regimen combining rituximab, high-dose methotrexate, thiotepa, and berubicin, an anthracycline developed to penetrate the blood–brain barrier. Four patients completed all planned cycles and achieved an early partial response, while two subsequently underwent autologous stem-cell transplantation. No protocol-defined dose-limiting toxicity or berubicin dose reduction was observed. However, all patients experienced severe adverse events, predominantly hematologic. The study was terminated prematurely because berubicin became unavailable. Although the small case series precludes definitive conclusions, these observations provide preliminary evidence of MBTRIX feasibility and may support further evaluation in larger studies.

## 1. Introduction

Primary central nervous system lymphoma (PCNSL) is a rare and aggressive extranodal lymphoma confined to the central nervous system, most commonly of diffuse large B-cell histology [1,2]. High-dose methotrexate (HD-MTX)–based polychemotherapy remains the backbone of first-line treatment, and in fit, transplant-eligible patients, intensive induction followed by thiotepa-based high-dose chemotherapy and autologous stem-cell transplantation (ASCT) represents an established consolidation strategy [3,4,5,6,7]. Despite these advances, a substantial proportion of patients experience refractory disease or relapse, and treatment remains particularly challenging in older or functionally impaired patients [1,3,8,9].

The efficacy of systemic treatment in CNS lymphoma is limited in part by the blood–brain barrier, which restricts CNS exposure to many otherwise active anticancer agents. Berubicin (WP744; RTA 744) is a doxorubicin analog developed to improve CNS penetration. It acts as a topoisomerase II poison and has demonstrated reduced susceptibility to major multidrug-efflux transporters in preclinical models [10,11,12]. Conventional anthracycline-containing regimens have not established a clinically meaningful role in PCNSL, largely because of inadequate CNS exposure [13,14]. Early clinical experience with berubicin has been reported mainly in patients with primary brain tumors and remains limited, with much of the available evidence derived from early-phase studies and conference reports [12,15,16,17].

MBTRIX was developed as an investigational CNS-directed regimen combining rituximab, high-dose methotrexate, thiotepa, and berubicin, with the intention of preserving the possibility of thiotepa-based ASCT in responding patients. In the prospective BER-PUM study, berubicin was administered once per 21-day cycle using a protocol-defined dose-escalation strategy. The initially planned study population comprised 15 patients. Recruitment was terminated prematurely because the investigational medicinal product became unavailable, and the planned dose-escalation phase was not completed.

We therefore report the clinical courses of five patients with isolated CNS lymphoma treated prospectively with MBTRIX within the BER-PUM study, focusing on treatment delivery, toxicity, tumor response, and the ability to proceed to consolidative ASCT.

## 2. Detailed Case Description

### 2.1. Clinical and Treatment Framework

The five patients described in this case series were treated prospectively within the investigator-initiated BER-PUM study, an open-label, single-center phase Ib/II trial conducted at the Department of Hematology and Transplantology, Pomeranian Medical University in Szczecin, Poland (protocol BER-PUM-01; EudraCT 2021-006028-41). Patients were enrolled between 6 September 2023 and 14 March 2025. The protocol was approved by the Bioethics Committee of the Pomeranian Medical University in Szczecin (approval no. KB-0012/52/2021, dated 20 December 2021), and all participants provided written informed consent. Recruitment was terminated prematurely because the investigational medicinal product became unavailable, preventing completion of the planned dose-escalation phase.

**Figure 1:**
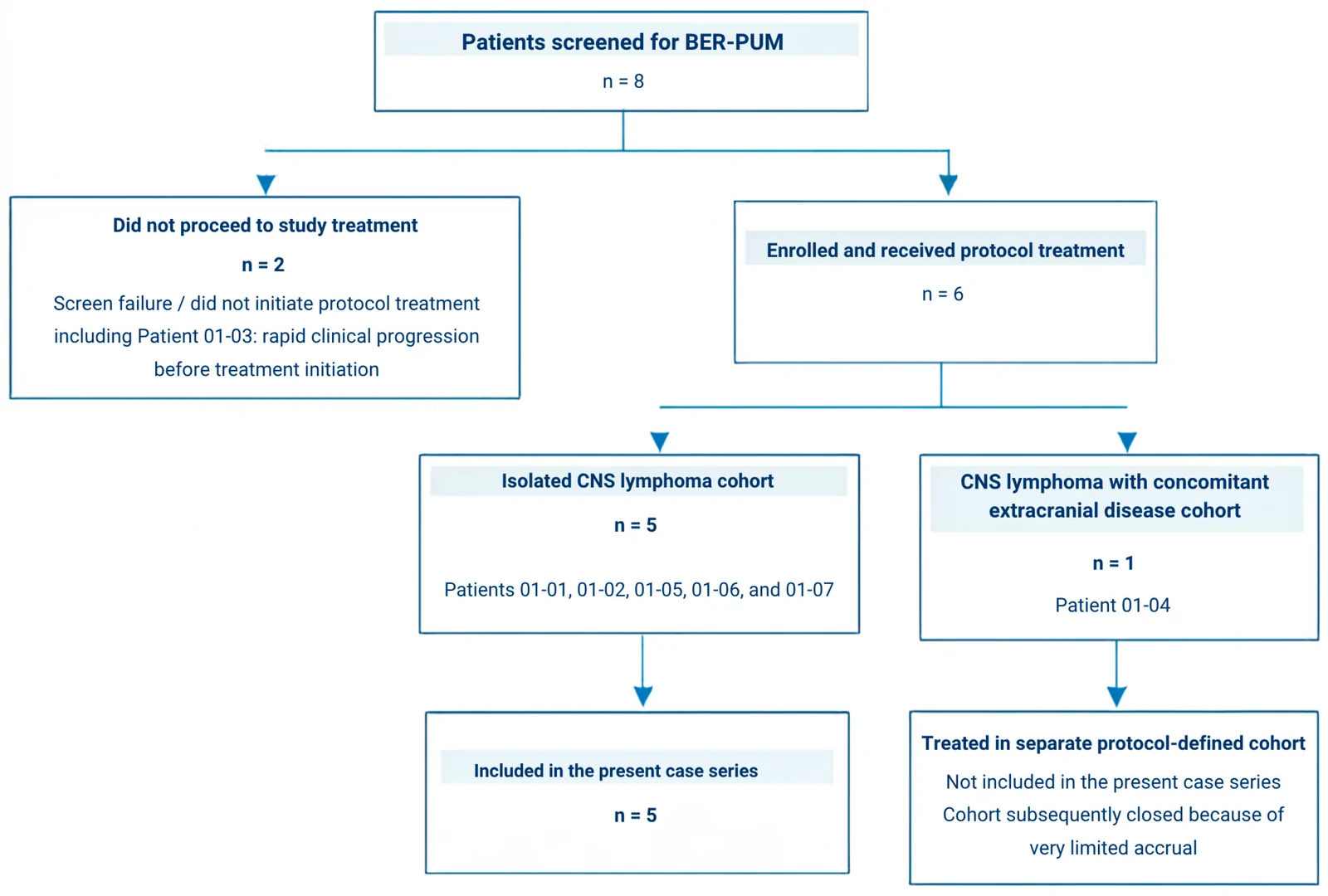
Participant flow diagram for the BER-PUM study and the present case series.

Overall, eight patients were screened for BER-PUM. Six were enrolled and received protocol treatment, whereas two were classified as screen failures and did not initiate study treatment. Patient identifiers reflect sequential study numbering within the original BER-PUM protocol rather than consecutive inclusion in the present case series. Patient 01-03 did not initiate study treatment because of rapid clinical progression before the first treatment cycle. Patient 01-04 received protocol treatment within a separate protocol-defined cohort for CNS lymphoma with concomitant extracranial disease and was therefore not included in the present report, which was restricted to patients with isolated CNS lymphoma. This second protocol cohort was subsequently closed because of very limited accrual. Patient disposition is summarized in Figure 1.

Patients received up to four 21-day cycles of MBTRIX, comprising rituximab 375 mg/m^2^ on day 1, high-dose methotrexate 3.5 g/m^2^ on day 2, berubicin on day 3 as a 2 h intravenous infusion, and thiotepa 30 mg/m^2^ on day 4. Berubicin was administered once per cycle using an accelerated titration design with one-patient cohorts and both between-patient and intrapatient dose escalation. The starting dose was 4.8 mg/m^2^, and each subsequent dose level increased by 1.92 mg/m^2^, corresponding to 40% of the starting dose. Dose escalation was guided by protocol-defined dose-limiting toxicity (DLT) assessment and ongoing safety evaluation as patients proceeded through successive treatment cycles. According to the protocol, a DLT comprised a clinically significant adverse event or laboratory abnormality occurring within the defined DLT evaluation period, except for events clearly attributable to disease progression, the underlying disease, intercurrent illness, or concomitant medications. Nonhematologic or extramedullary DLTs generally comprised grade ≥ 3 toxicities, with prespecified exceptions for expected or clinically manageable events. Hematologic DLT was defined as failure of neutrophil and/or platelet recovery within 6 weeks after the last study-drug dose in the absence of disease. Thus, transient severe cytopenias, including grade 4 neutropenia followed by appropriate hematologic recovery, did not by themselves meet the protocol definition of hematologic DLT. Safety was monitored throughout treatment, and continuation and escalation were additionally subject to protocol-defined hematologic recovery and treatment-interruption criteria. No protocol-defined DLT occurred in the treated patients. Because recruitment ended before completion of dose escalation, neither the maximum tolerated dose nor the recommended phase 2 dose was established. The highest dose administered, 18.2 mg/m^2^, therefore represents the highest protocol-defined dose level reached in this limited cohort and should not be interpreted as an established maximum tolerated dose or tolerable dose range.

Treatment response was assessed after every two cycles and at the end of treatment using contrast-enhanced brain MRI and the International Primary CNS Lymphoma Collaborative Group response criteria [18]. All patients had been referred to our center from other institutions and had heterogeneous diagnostic and pre-study clinical pathways. Dexamethasone was not part of the BER-PUM treatment regimen. Three of the five patients had not received dexamethasone, whereas in the remaining two patients corticosteroids had been administered transiently before study enrollment in referring neurology or neurosurgery departments, according to neurological symptoms and cerebral edema, while diagnostic work-up and histopathological confirmation were pending. Corticosteroid treatment had been discontinued before enrollment, and no patient received dexamethasone during protocol treatment or at the protocol-defined response assessments.

Cerebrospinal fluid assessment by cytology and flow cytometry was performed at baseline and before Cycle 3, corresponding to the first response assessment after two treatment cycles, with additional assessment when clinically indicated. Ophthalmologic examination was performed according to the study protocol when clinically indicated. Baseline and post-treatment MRI examinations were reviewed locally by an experienced radiologist together with the Investigator; no independent central imaging review was performed.

Patients achieving at least a partial response and remaining eligible for consolidation could undergo peripheral blood stem-cell collection followed by thiotepa-containing high-dose therapy and autologous stem-cell transplantation. Adverse events were graded according to CTCAE version 5.0. Safety monitoring commenced after informed consent and continued through the end-of-treatment follow-up visit.

### 2.2. Patient 01-01

Patient 01-01 was a 67-year-old woman with newly diagnosed primary CNS lymphoma. She initially presented with headache, gait imbalance with a tendency to fall to the left, and progressive left-sided neurological deficits, including homonymous hemianopia and hemiparesis. Brain MRI demonstrated multifocal CNS involvement, predominantly in the right cerebral hemisphere, with the largest contrast-enhancing lesion measuring 36 × 17 mm in the medial right parietal lobe and an additional approximately 17 mm lesion involving the deep cerebral structures; less prominent abnormalities were also present in the pons and right cerebellar hemisphere.

A neuronavigation-guided brain biopsy was performed on 7 August 2023. Histopathological examination revealed infiltration by diffuse large B-cell lymphoma (DLBCL) of non-germinal-center B-cell (non-GCB) phenotype, with a Ki-67 proliferation index of approximately 90%. The final histopathological diagnosis was communicated to the patient on 4 September 2023.

MBTRIX treatment was initiated on 12 September 2023 (Cycle 1, Day 1). The patient completed all four planned MBTRIX cycles, with intrapatient berubicin dose escalation from 4.8 to 6.7, 8.6, and 10.6 mg/m^2^ across Cycles 1–4. Before initiation of Cycle 3, she developed symptoms of a urinary tract infection, which completely resolved after antibiotic therapy. No berubicin dose reduction or omission was required. MRI after two cycles demonstrated a partial response, which represented her best response during MBTRIX treatment. Despite completion of all four cycles, the end-of-treatment assessment demonstrated progressive disease, precluding consolidative autologous stem-cell transplantation. At the last documented follow-up on 21 February 2024, further disease progression was confirmed. The patient subsequently experienced continued clinical deterioration and died on 18 March 2024, approximately one month after the last documented progression assessment.

The principal treatment-emergent hematologic toxicity was grade 3 anemia, assessed as probably related to berubicin. Additional adverse events included grade 2 urinary tract infection, grade 1 gastropathy, and grade 1 hemorrhoids, all assessed as unrelated to berubicin. None of these events required modification of berubicin dosing.

### 2.3. Patient 01-02

Patient 01-02 was a 54-year-old man with newly diagnosed primary CNS lymphoma of diffuse large B-cell histology. Diagnostic evaluation was initiated because of a gradually progressive left-sided motor deficit with increasing spasticity of the left upper limb. Neurologically, he had predominantly left-sided spastic hemiparesis, more pronounced in the upper limb, with impaired gait. Baseline brain MRI demonstrated a contrast-enhancing lesion in the right cerebral peduncle measuring approximately 1.5 × 1.3 × 2.2 cm. A transcranial brain biopsy was performed on 8 November 2023, establishing the diagnosis of PCNSL. The final histopathological diagnosis was available on 23 November 2023.

At study enrollment, his ECOG performance status was 2, and the IELSG score was 4.

MBTRIX treatment was initiated on 9 December 2023 (Cycle 1, Day 1). He completed all four planned MBTRIX cycles, with intrapatient berubicin dose escalation from 6.7 to 8.6, 10.6, and 12.5 mg/m^2^ across Cycles 1–4. No berubicin dose reduction or omission was required. MRI after two cycles demonstrated a partial response. The first treatment cycle was complicated by infectious events, including an *E. coli* urinary tract infection, oral candidiasis, and fever of unknown origin (FUO). The fourth cycle was also complicated by an *E. coli* urinary tract infection. At the end-of-treatment assessment on 28 March 2024, the enhancing component of the lesion in the right cerebral peduncle had further regressed; however, new CNS lesions had emerged, resulting in an overall assessment of progressive disease. Consequently, the patient was not eligible for consolidative autologous stem-cell transplantation.

The principal treatment-emergent toxicity was grade 3 neutropenia, assessed as probably related to berubicin. During a prolonged interval between treatment cycles, the patient also developed a grade 4 pulmonary embolism that met clinical seriousness criteria but was assessed as unrelated to the investigational product. Neither event required permanent discontinuation of berubicin because of toxicity.

Following progression after MBTRIX, the patient received salvage treatment with tafasitamab and lenalidomide. Despite subsequent therapy, the lymphoma continued to progress and was associated with further clinical deterioration, leading to the institution of palliative care.

At the Hematology Clinic assessment on 10 June 2024, continued disease progression and clinical deterioration were documented, and the patient was subsequently transferred to hospice care. He died on 11 June 2024.

### 2.4. Patient 01-05

Patient 01-05 was a 69-year-old man with newly diagnosed primary CNS lymphoma. Diagnostic evaluation was initiated because of speech disturbance and progressive left-sided hemiparesis. Brain MRI demonstrated a contrast-enhancing corticosubcortical lesion at the right frontoparietal junction measuring approximately 18 × 28 × 17 mm, associated with marked surrounding edema and mild mass effect. A brain biopsy was performed on 2 February 2024, establishing the diagnosis of PCNSL. The final histopathological diagnosis was available in March 2024. At study enrollment, his ECOG performance status was 2, and the IELSG score was 4.

MBTRIX treatment was initiated on 18 March 2024 (Cycle 1, Day 1). He completed all four planned MBTRIX cycles, with intrapatient berubicin dose escalation from 8.6 to 10.6, 12.5, and 14.4 mg/m^2^ across Cycles 1–4. During treatment, grade 2 renal impairment developed and was attributed to high-dose methotrexate. Consequently, the methotrexate dose was reduced by 25% in Cycles 2–4, while the planned berubicin escalation was maintained. MRI after two cycles demonstrated a partial response, which was maintained at the end-of-treatment assessment.

Peripheral blood stem-cell collection was successful, and the patient proceeded to autologous stem-cell transplantation after BuTT conditioning. Hematopoietic recovery of both neutrophils and platelets was achieved by day + 13. Post-transplant MRI demonstrated complete response, which remained radiologically confirmed at 12 months after ASCT.

The principal treatment-emergent toxicity was grade 4 neutropenia, assessed as probably related to berubicin. Grade 2 paroxysmal atrial fibrillation was also assessed as probably related to berubicin and resolved with medical management without requiring interruption or dose reduction. Additional events included grade 2 sore throat and grade 2 weakness, both assessed as possibly related to study treatment, and the aforementioned grade 2 renal impairment attributed to high-dose methotrexate. No infectious complications occurred during MBTRIX treatment. No berubicin dose reduction, omission, or discontinuation was required because of toxicity.

At the most recent follow-up on 15 July 2026, the patient was in good general condition, with no evidence of central nervous system lymphoma recurrence.

### 2.5. Patient 01-06

Patient 01-06 was a 71-year-old man with a history of right testicular diffuse large B-cell lymphoma of non-germinal-center B-cell phenotype, diagnosed in 2018. He underwent orchiectomy followed by eight cycles of R-CHOP and three administrations of intrathecal methotrexate, cytarabine, and dexamethasone, achieving complete metabolic remission. Nearly five years later, he developed progressive disorientation, memory impairment, and loss of independence. Brain MRI demonstrated a large infiltrative lesion involving the corpus callosum, measuring approximately 86 × 37 × 29 mm, with bilateral extension, contrast enhancement, diffusion restriction, and surrounding edema. A brain biopsy was performed on 7 March 2024, and the final histopathological diagnosis was established on 22 March 2024. Six years elapsed between the diagnosis of DLBCL and the relapse involving the CNS. Cerebrospinal fluid analysis demonstrated a clonal CD19+/CD20+ B-cell population with lambda light-chain restriction, while systemic staging showed no evidence of extracranial lymphoma involvement, supporting the diagnosis of isolated secondary CNS relapse. At study enrollment, his ECOG performance status was 2.

MBTRIX treatment was initiated on 6 April 2024 (Cycle 1, Day 1). The patient received two MBTRIX cycles, with berubicin administered at 10.6 and 12.5 mg/m^2^ in Cycles 1 and 2, respectively. No infectious complications occurred during MBTRIX treatment. MRI after two cycles demonstrated progressive disease, and study treatment was discontinued. He subsequently experienced rapid neurological deterioration with decline in performance status to ECOG 4. Given the extent of disease progression and marked functional deterioration, further intensive lymphoma-directed therapy was not pursued. At the last documented clinical assessment on 22 May 2024, the patient was transferred to a palliative care unit. Hospice-based supportive care was subsequently continued. He died on 5 June 2024.

Adverse events during MBTRIX included grade 2 anemia and grade 1 thrombocytopenia, both assessed as probably related to berubicin, and grade 2 fever of uncertain origin, assessed as possibly related to study treatment. He also developed grade 3 cognitive disturbance, which was assessed as unrelated to study treatment and attributed to radiologically confirmed lymphoma progression.

### 2.6. Patient 01-07

Patient 01-07 was a 64-year-old man with newly diagnosed primary CNS lymphoma of diffuse large B-cell histology. The disease involved the left temporal region and was associated with secondary seizures. Brain imaging demonstrated a left temporal lesion accompanied by a subacute intralesional or perilesional hemorrhagic component measuring approximately 30 × 21 mm, with a residual peripheral tumor component and limited surrounding edema. The patient underwent left-sided temporoparietal neurosurgical intervention with tissue sampling on 26 March 2024. Histopathological examination dated 28 March 2024 demonstrated a diffuse infiltrate of large B-cell malignant lymphoma. These findings established the diagnosis of PCNSL. The patient was subsequently hospitalized in the study unit from 11 April 2024. At study enrollment, his ECOG performance status was 1, and the IELSG score was 1.

MBTRIX treatment was initiated on 23 April 2024 (Cycle 1, Day 1). He completed all four planned MBTRIX cycles, with intrapatient berubicin dose escalation from 12.5 to 14.4, 16.3, and 18.2 mg/m^2^ across Cycles 1–4. No infectious complications occurred during MBTRIX treatment. No berubicin dose reduction, omission, or discontinuation was required. MRI after two cycles demonstrated a partial response, which was maintained at the end-of-treatment assessment. Peripheral blood stem-cell collection was successful, and the patient proceeded to autologous stem-cell transplantation after BuTT conditioning.

During the transplant hospitalization, the patient developed severe infectious and respiratory complications, including *Stenotrophomonas* pneumonia and evidence of viral pneumonitis; CMV DNA was also detected in bronchoalveolar lavage. His clinical course progressed to respiratory failure and multiorgan dysfunction. At the last documented clinical assessment on 27 August 2024, he was transferred to the intensive care unit. Despite intensive supportive treatment, he died on 20 September 2024. These post-transplant complications were assessed as unrelated to berubicin.

During MBTRIX treatment, the principal adverse events were hematologic: grade 4 neutropenia, grade 3 anemia, and grade 3 thrombocytopenia, all assessed as probably related to berubicin. He also experienced grade 1 conjunctivitis, assessed as unrelated. Despite these toxicities, he completed all four planned MBTRIX cycles and the protocol-defined intrapatient berubicin escalation.

### 2.7. Integrated Clinical Course and Safety of the Case Series

Five patients received at least one dose of MBTRIX and constituted the treated case series. Four patients had PCNSL, and one had an isolated secondary CNS relapse of DLBCL. The median age was 67 years (range, 54–71), four patients were male, and four had an ECOG performance status of 2. Baseline characteristics are summarized in Table 1.

Four of five patients completed all four planned MBTRIX cycles; one discontinued treatment after two cycles because of radiologically confirmed progressive disease. Four patients achieved a partial response after two cycles. Among these initial responders, two subsequently developed progressive disease before transplantation, whereas two maintained a partial response through the end-of-treatment assessment and proceeded to ASCT. Peripheral blood stem-cell collection was successfully performed in three patients. Individual berubicin dose escalation, treatment modifications, responses, and subsequent clinical outcomes are summarized in Table 2.

All five patients experienced at least one grade 3 or higher adverse event, and four experienced at least one grade 3 or higher event assessed as probably related to berubicin. Grade 3 or higher neutropenia occurred in three patients, anemia in two, and thrombocytopenia in one. One patient developed grade 2 renal impairment attributed to high-dose methotrexate, requiring a 25% methotrexate dose reduction without modification of berubicin. Another experienced grade 2 paroxysmal atrial fibrillation assessed as probably related to berubicin. Protocol-defined cardiac surveillance included serial ECG, echocardiographic assessment of left ventricular ejection fraction, troponin, and NT-proBNP. No clinically meaningful decline in LVEF, relevant increase in cardiac injury biomarkers, or new clinically significant ECG abnormalities were observed during MBTRIX treatment. Patients 01-02 developed a grade 4 pulmonary embolism that met seriousness criteria but was assessed as unrelated to the investigational product. No protocol-defined dose-limiting toxicity occurred, and no berubicin dose was reduced, omitted, or discontinued because of toxicity. Individual adverse events are presented in Table 3.

Study recruitment ended before completion of the planned dose-escalation phase because berubicin became unavailable. Consequently, the maximum tolerated dose and recommended phase 2 dose were not established.

## 3. Discussion

This prospective case series provides initial clinical experience with MBTRIX, a modified MATRix-based regimen in which cytarabine was replaced by berubicin, in five patients with isolated CNS lymphoma. Four patients achieved a partial response after two cycles, but durable disease control was not universal: two subsequently progressed before consolidation, whereas two maintained a partial response through the end of treatment and proceeded to thiotepa-based ASCT. One of the transplanted patients subsequently achieved a complete response maintained at 12 months after ASCT. These findings indicate that early radiological responses can occur with MBTRIX, but they should be interpreted cautiously. Because rituximab, high-dose methotrexate, and thiotepa all have established activity in CNS lymphoma and no comparator group was available, the independent contribution of berubicin to the observed responses cannot be determined. Accordingly, the radiological responses observed in this study should be interpreted as activity of the MBTRIX combination rather than as evidence of berubicin-specific efficacy.

The rationale for incorporating berubicin into a CNS-directed regimen is based on its pharmacologic properties and prior clinical development. Berubicin is a 4′-O-benzylated doxorubicin analog with reduced susceptibility to major multidrug-efflux transporters. In preclinical models, WP744 demonstrated greater intracellular accumulation and cytotoxicity than doxorubicin or daunorubicin in cells expressing P-glycoprotein, MRP1, and BCRP [10]. Additional preclinical and early clinical-development reports suggested penetration and retention within brain and brain-tumor tissue [11,15], although direct evidence of therapeutically relevant CNS exposure in humans remains limited. Previous clinical experience has predominantly involved recurrent primary brain tumors. In an early phase I program, berubicin was administered on three consecutive days every 21 days, with a reported maximum tolerated dose of 7.5 mg/m^2^ per day; severe hematologic toxicity, particularly neutropenia and thrombocytopenia, represented the principal dose-limiting toxicities [15,16]. These dose levels are not directly comparable with MBTRIX because berubicin was administered once per cycle with intrapatient dose escalation. More recently, the randomized CNS-201 study in recurrent glioblastoma showed no overall-survival advantage over lomustine, although partial responses occurred with berubicin and myelosuppression remained an important toxicity [17]. These data provide biological and safety context for the present cases but cannot be extrapolated directly to CNS lymphoma.

The ability to deliver intensive therapy is an important consideration in PCNSL, particularly because many patients are older or have impaired performance status. High-dose methotrexate remains the backbone of first-line treatment, and MATRix is among the best-established induction regimens for fit patients considered candidates for intensive consolidation [3,4,19]. In IELSG32, MATRix improved disease-control outcomes but was associated with substantial grade 4 hematologic toxicity and treatment-related mortality [4]. Real-world experience further illustrates the challenges of delivering this intensity outside highly selected trial populations. In an international routine-practice cohort, the median ECOG performance status was 2 and treatment-related mortality was approximately 6%, while patients who did not meet IELSG32 eligibility criteria received lower treatment intensity and had inferior outcomes [20]. Similarly, in the NiHiL registry, only 59% of trial-eligible patients completed MATRix, with discontinuation frequently related to toxicity or infection and treatment-related mortality of 8% [21]. Against this background, completion of all four planned MBTRIX cycles by four of five patients is clinically relevant, particularly given a median age of 67 years and ECOG performance status of 2 in four patients. However, this observation should not be interpreted as evidence of improved tolerability compared with MATRix, because MBTRIX is a different regimen in which high-dose cytarabine was replaced by berubicin. Furthermore, rituximab was administered once per cycle in the MBTRIX regimen, unlike the twice-per-cycle administration in the MATRIX regimen.

The inclusion of one patient with isolated secondary CNS relapse warrants separate consideration. PCNSL and secondary CNS lymphoma share the challenge of achieving effective CNS drug exposure but differ in natural history, prior systemic treatment, and risk of extracranial disease. Prospective evidence in secondary CNS lymphoma is represented most prominently by the MARIETTA study, which evaluated sequential MATRix and R-ICE followed by thiotepa-containing ASCT in a heterogeneous population that included CNS involvement at diagnosis, isolated CNS relapse, and concomitant systemic and CNS relapse [22]. Consequently, these data do not provide a direct comparator for the single patient with isolated secondary CNS recurrence described here. Contemporary treatment strategies generally incorporate high-dose methotrexate–based CNS-directed therapy and consider thiotepa-based ASCT in responding, eligible patients, while evidence specific to isolated CNS relapse remains limited [23,24].

Toxicity across the five cases was predominantly hematologic. Grade 3 or higher adverse events occurred in all patients, with severe neutropenia, anemia, and thrombocytopenia constituting the principal events assessed as probably related to berubicin. This pattern is consistent with previous clinical experience with berubicin [15,16], although substantial hematologic toxicity is also expected with intensive multiagent CNS lymphoma therapy, including MATRix [4,20]. Consequently, attribution of individual cytopenias specifically to berubicin is difficult. However, the absence of protocol-defined DLT should not be interpreted as evidence of overall tolerability, particularly given the small cohort, incomplete dose escalation, and the occurrence of grade ≥ 3 adverse events in all patients. Among non-hematologic events, grade 2 renal impairment in one patient was attributed to high-dose methotrexate and resulted in methotrexate dose reduction without modification of berubicin. Grade 2 paroxysmal atrial fibrillation in another patient was assessed as probably related to berubicin, whereas a grade 4 pulmonary embolism in a different patient was assessed as unrelated to the investigational product. Anthracyclines are associated with acute cardiac rhythm disturbances, while CNS lymphoma itself and its treatment are associated with multiple risk factors for venous thromboembolism [25,26]. Given the small number of patients and the occurrence of single cardiac and thromboembolic events, no specific cardiac or thrombotic safety signal can be inferred from this series.

Two patients maintained a partial response through the end of MBTRIX treatment and proceeded to thiotepa-containing ASCT, with markedly different outcomes. One converted from PR to CR after transplantation and remained in MRI-confirmed remission at 12 months after ASCT, whereas the other died during the transplant hospitalization from severe infectious and respiratory complications leading to multiorgan failure; these complications were assessed as unrelated to berubicin. These contrasting courses illustrate both the potential for durable disease control after successful consolidation and the substantial risks associated with high-dose therapy. Thiotepa-based ASCT is a well-established consolidation strategy for fit patients with PCNSL, supported by prospective and randomized studies [27,28,29,30]. Most recently, the phase III IELSG43 trial demonstrated superior disease control with high-dose chemotherapy followed by ASCT compared with non-myeloablative consolidation after MATRix, further strengthening the role of ASCT in eligible patients, while also confirming the clinically relevant risks of severe infection and treatment-related mortality [28]. Real-world data similarly support the effectiveness of this approach in appropriately selected patients [31].

The present report has several important limitations. Only five patients were treated at a single center; the cohort included both primary and secondary CNS lymphoma, and there was no control group, precluding assessment of relative efficacy or toxicity. Recruitment ended prematurely because the investigational medicinal product became unavailable, preventing completion of the planned dose-escalation program; consequently, neither a maximum tolerated dose nor a recommended phase 2 dose was established. Because berubicin was escalated within individual patients across successive treatment cycles, toxicity emerging at higher dose levels cannot be attributed unequivocally to the current dose rather than to cumulative exposure to berubicin and the other components of MBTRIX. Both dose-related and cumulative toxicity were relevant to ongoing safety assessment and could limit further escalation if protocol-defined criteria were met. However, given the very small number of patients exposed to each higher dose level, the study cannot define a reliable dose–toxicity relationship or separate cumulative from dose-dependent toxicity. In addition, pharmacokinetic data were incomplete and did not permit meaningful interpretation of systemic or CNS berubicin exposure. No further pharmacokinetic analysis within the BER-PUM study is currently planned. Within these constraints, the present case series provides prospective clinical experience with berubicin incorporated into a high-dose methotrexate–based regimen and documents treatment delivery, toxicity, radiological response, progression, and transition to transplantation. Intrapatient berubicin escalation was technically feasible in the treated patients and did not itself lead to dose reduction, omission, or discontinuation. Further prospective evaluation would be required to define the safety profile, pharmacokinetics, and independent contribution of berubicin to CNS lymphoma treatment.

## 4. Conclusions

In this prospective case series derived from the prematurely terminated phase Ib/II BER-PUM study, once-per-cycle berubicin was incorporated into the MBTRIX regimen with protocol-defined dose escalation in five patients with isolated CNS lymphoma. Early radiological responses were observed, and two patients proceeded to consolidative ASCT; however, the small uncontrolled cohort, incomplete dose escalation, and lack of interpretable pharmacokinetic data preclude conclusions regarding the independent safety or efficacy of berubicin. These observations support further prospective evaluation of berubicin within CNS-directed treatment strategies.

## Figures and Tables

**Table 1:** Baseline Characteristics of Patients with Isolated CNS Lymphoma.

Characteristic	Study Population, *n* = 5
Age, years, median (range)	67 (54–71)
Male sex, *n*	4
ECOG performance status, *n*	
-ECOG 1	1
-ECOG 2	4
Lymphoma category, *n*	
-Primary central nervous system lymphoma	4
-Isolated secondary central nervous system relapse	1
Diffuse large B-cell lymphoma histology, *n*	5
IELSG prognostic score, median (range) *	4 (1–4)
High-risk IELSG category, *n*/N *	3/4

* Calculated only for patients with primary CNS lymphoma. Abbreviations: ECOG, Eastern Cooperative Oncology Group; IELSG, International Extranodal Lymphoma Study Group.

**Table 2:** Individual Treatment Exposure, Tumor Response, and Clinical Outcomes in the Treated Cohort.

Patient	Completed Cycles, *n*	Berubicin Dose by Cycle, mg/m^2^	Treatment Modification	Response After Two Cycles	Response at End of Treatment	Best Response During MBTRIX Treatment	Proceeded to ASCT	Subsequent Clinical Course
**01-01**	4	C1: 4.8; ↓ C2: 6.7; ↓ C3: 8.6; ↓ C4: 10.6	None	PR	PD	PR	No	Disease progression before ASCT; death approximately 1 months after MRI-confirmed progression
**01-02**	4	C1: 6.7; ↓ C2: 8.6; ↓ C3: 10.6; ↓ C4: 12.5	None	PR	PD	PR	No	Disease progression before ASCT
**01-05**	4	C1: 8.6; ↓ C2: 10.6; ↓ C3: 12.5; ↓ C4: 14.4	Methotrexate dose reduced by 25% in cycles 2–4 because of grade 2 renal impairment	PR	PR	PR	Yes	MRI-confirmed CR after ASCT, maintained at 12 months after ASCT
**01-06**	2	C1: 10.6; ↓ C2: 12.5	Treatment discontinued because of PD	PD	Not applicable	PD	No	Rapid neurological deterioration, transition to palliative care, and death
**01-07**	4	C1: 12.5; ↓ C2: 14.4; ↓ C3: 16.3; ↓ C4: 18.2	None	PR	PR	PR	Yes	Death during the ASCT hospitalization from severe infectious, respiratory, and multiorgan complications assessed as unrelated to berubicin

Berubicin doses are presented in mg/m^2^ by consecutive cycle; arrows indicate intrapatient dose escalation. Each subsequent dose level represented an increase of 1.92 mg/m^2^, corresponding to 40% of the starting dose. Abbreviations: ASCT, autologous stem-cell transplantation; C, treatment cycle; CR, complete response; MRI, magnetic resonance imaging; PD, progressive disease; PR, partial response.

**Table 3:** Adverse Events Observed during MBTRIX Study Treatment.

Patient	Adverse Event	Maximum CTCAE Grade	Relationship or Attribution
01-01	Anemia	3	Probably related to berubicin
	Urinary tract infection	2	Unrelated
	Gastropathy	1	Unrelated
	Hemorrhoids	1	Unrelated
01-02	Neutropenia	3	Probably related to berubicin
	Pulmonary embolism ^1^	4	Unrelated
01-05	Neutropenia	4	Probably related to berubicin
	Renal impairment	2	Unrelated to berubicin; attributed to high-dose methotrexate
	Paroxysmal atrial fibrillation	2	Probably related to berubicin
	Sore throat	2	Possibly related to study treatment
	Weakness	2	Possibly related to study treatment
01-06	Anemia	2	Probably related to berubicin
	Thrombocytopenia	1	Probably related to berubicin
	Fever of uncertain origin	2	Possibly related to study treatment
	Cognitive disturbance ^2^	3	Unrelated; attributed to lymphoma progression
01-07	Neutropenia	4	Probably related to berubicin
	Anemia	3	Probably related to berubicin
	Thrombocytopenia	3	Probably related to berubicin
	Conjunctivitis	1	Unrelated

^1^ The grade 4 pulmonary embolism occurred during a prolonged interval between treatment cycles, met clinical seriousness criteria, and was assessed as unrelated to the investigational product. ^2^ Cognitive disturbance was attributed to radiologically confirmed lymphoma progression rather than study treatment. Abbreviations: CTCAE, Common Terminology Criteria for Adverse Events.

## Data Availability

The data presented in this study are available on request from the corresponding author due to privacy issues.
